# Spatio-temporal dynamic of the COVID-19 epidemic and the impact of imported cases in Rwanda

**DOI:** 10.1186/s12889-023-15888-1

**Published:** 2023-05-23

**Authors:** Muhammed Semakula, François Niragire, Sabin Nsanzimana, Eric Remera, Christel Faes

**Affiliations:** 1 I-BioStat, Hasselt University, Hasselt, Belgium; 2 College of Business and Economics, Centre of excellence in Data Science, Bio-statistics, University of Rwanda, Kigali, Kigali, Rwanda; 3 Rwanda Biomedical Centre, Ministry of Health, Kigali, Rwanda; 4 Department of Applied Statistics, University of Rwanda, Kigali, Kigali, Rwanda

**Keywords:** COVID-19, Spatio-temporal models, Epidemiology

## Abstract

**Introduction:**

Africa was threatened by the coronavirus disease 2019 (COVID-19) due to the limited health care infrastructure. Rwanda has consistently used non-pharmaceutical strategies, such as lockdown, curfew, and enforcement of prevention measures to control the spread of COVID-19. Despite the mitigation measures taken, the country has faced a series of outbreaks in 2020 and 2021.

In this paper, we investigate the nature of epidemic phenomena in Rwanda and the impact of imported cases on the spread of COVID-19 using endemic-epidemic spatio-temporal models. Our study provides a framework for understanding the dynamics of the epidemic in Rwanda and monitoring its phenomena to inform public health decision-makers for timely and targeted interventions.

**Results:**

The findings provide insights into the effects of lockdown and imported infections in Rwanda’s COVID-19 outbreaks. The findings showed that imported infections are dominated by locally transmitted cases. The high incidence was predominant in urban areas and at the borders of Rwanda with its neighboring countries. The inter-district spread of COVID-19 was very limited due to mitigation measures taken in Rwanda.

**Conclusion:**

The study recommends using evidence-based decisions in the management of epidemics and integrating statistical models in the analytics component of the health information system.

**Supplementary Information:**

The online version contains supplementary material available at 10.1186/s12889-023-15888-1.

## Introduction

Due to limited health care infrastructure in most African countries, Africa was severely threatened by the coronavirus disease COVID-19 [1]. Worldwide, up to 349 million COVID-19 cases were reported by December 2021, with around 6 million reported in Africa. As of December 19th, 2021, in Rwanda the total number of COVID-19 cases in the population was 102,231, with females accounting for over 51% of the cases. The majority of cases (80%) occurred in individuals under the age of fifty. On average, approximately 1000 PCR tests were conducted per day, with a COVID-19 recovery rate of 98%. The case fatality rate was 1.1%, with 1125 recorded deaths, of which the majority were males (53%) [2]. Rwanda’s swift, methodical, and all-encompassing strategy to combat the COVID-19 outbreak has been lauded for its effectiveness. Despite the inevitable economic consequences, the country’s GDP declined by 39.1% when compared to a hypothetical scenario in which the pandemic did not occur during the same period. The pace and extent of Rwanda’s economic recovery will be contingent on various factors, including the resumption of international travel [3].

By the end of 2021, concerns were growing over a third and fourth wave of infections, with Africa’s COVID-19 vaccine rollout advancing slower than the rest of the world. Fortunately, the continent also has particular experience in controlling pandemics using effective surveillance strategies to prevent the spread of diseases [4]. Even before any African country had reported a COVID-19 case, Rwanda had already established an early warning system in January 2020. This system included temperature screening at airports and recording travelers at points of entry to enable public health tracing [5]. Strong measures such as imposing lockdown and systematic contact tracing to interrupt the chain of transmission were implemented [6]. We have provided in supplementary map of districts of Rwanda with names,location of airport and key point of entry. Rwanda uses cellphone tower data to augment contact tracing efforts [7], conducts decentralized contact tracing at community level, and uses geospatial mapping to monitor the spread of the disease.

The spread of the disease naturally exhibits spatio-temporal interaction, as an infectious individual may cause secondary cases by transmitting the infectious agent to nearby susceptible individuals [8]. Spatial and spatio-temporal models have been evolving rapidly in the last two decades, and their application to the health field improved the response to the pandemic [9]. The importance of geography in the study of disease transmission, like all-natural phenomena, answers to the first law of geography, stating that everything is related to everything else, but near things are more related than distant things [10]. The study of the secondary transmission pattern of COVID-19 in Rwanda showed that the spatial component is important to understand the spread of COVID-19 [6]. Held and al. proposed a framework for the statistical analysis of the number of cases of infectious diseases, describing the epidemic curve by an epidemic and endemic component. The model makes it possible to study the epidemic at a local scale and to investigate the spread amongst regions [11]. The epidemic-endemic model has been successfully applied for several infectious disease outbreaks, providing an adequate fit and reliable one-step-ahead predictions: e.g. dengue in China [12], measles in Cameroon [13], COVID-19 in Italy [10], UK [14] and Germany [15].

Despite several severely restrictive measures taken in Rwanda, local COVID-19 outbreaks continued to occur across the country. In the first quarter of the pandemic, imported cases were predominant. An imported case was defined as anyone who tested positive for COVID-19 at a point of entry into the country. All imported cases were quarantined and treated in government-supervised centers to reduce the transmission risk in the community. In addition, Rwanda imposed travel restrictions and closure of borders to limit imported cases. Despite those measures, more new cases resulted from local transmission compared with the amount of imported cases over time. Although the travel restrictions have clear benefits when there are no or few cases in a destination country, they are less effective once a country has a larger number of cases resulting from local community transmission [16].

The purpose of the present paper is to model the number of COVID-19 infections to understand the dynamics of the epidemic in Rwanda and to monitor epidemic phenomena to inform public health decision-makers, as this gives them the time to intervene in local public health systems with timely, evidence-based and targeted interventions in small areas. We investigate the nature of epidemic phenomena in Rwanda and the impact of imported cases on the spread of COVID-19 cases.

The study findings will contribute to inform public health policies and strategies aimed at curbing the spread of COVID-19 in the country. The findings of this study can also be used to guide future research and provide insights on modeling approaches of COVID-19 infections to inform adequate interventions to control the epidemic.

## Materials and methods

### Data source

The Rwanda Biomedical Center started disseminating the daily number of COVID-19 infections since the detection of the first case on 14th March 2020. In this paper, we used data from 14th March 2020 until 19th December 2021. Rwanda has a digitized COVID-19 data system, and everyone who tested positive from the laboratory system gets immediately reported to the public dashboard [6]. On 19th December 2021, Rwanda recorded a cumulative total of 102,231 cases of COVID-19. The COVID-19 cases were categorized into imported or locally transmitted cases. The lockdown data was collected based on the date and the geographic area, which could be a sector or a district. Information regarding the population of each district and the district shapefile of Rwanda were provided by National institute of statistics. The data used were accessed from the Rwanda Biomedical Center data hub at the link: https://gis.rbc.gov.rw/portal.

### Statistical analysis

The evolution of the number of daily infections is studied by means of a count model, which belongs to the family of Spatial Generalised Linear Mixed Models. Let $Y_{rt}$ be the number of reported cases in district $r = 1, \ldots , R$ during week $t= 1, \ldots , T$. An endemic-epidemic multivariate time-series model for infectious disease counts $Y_{rt}$ was proposed by Held, Höhle, and Hofmann [11] and later extended in a series of papers [8, 17, 18]. In its most common formulation, this model assumes that conditional on the past observations, the number of reported cases follows a negative binomial distribution with mean $\mu _{r,t}$, where

1 $$\begin{aligned} \mu _{r,t}=\lambda _{rt}Y_{r,t-1}+\phi _{rt}\sum \limits _{r'\ne r}\omega _{r^{'},r}Y _{r',t-1}+e_{r}\upsilon _{r,t}, \end{aligned}$$

and region-specific overdispersion parameters $\psi _{r}$. If $\psi _{r}$ > 0 the conditional variance of $Y_{r,t-1}$ is $\mu _{r,t}$ (1+$\psi _{r}\mu _{r,t}$), while if $\psi _{r}=$ 0 the negative binomial distribution reduces to a Poisson distribution with mean and variance equal to $\mu _{r,t}$. The three terms on the right-hand side of Eq. (1) correspond to the three components of the model: the epidemic within-region or auto-regressive component, the epidemic between-region or spatio-temporal component, and the endemic component.

The first component models the contribution of the temporal dynamics to the expected number of cases within region *r*. This component assumes a dependency on the number of cases observed in the previous week $(t- 1)$ in the region, with the coefficient $\lambda _{r} > 0$ quantifying the strength of dependence. The second component is the epidemic between-region component that models the contagion between neighbouring regions by including the incidence in the neighbouring regions $\sum _{r'\ne r}\omega _{r^{'},r}Y _{r',t-1}$, where $\omega _{r^{'},r}$ is positive if districts $r^{'}$ and *r* share a border and $\omega _{r^{'},r}$ is zero otherwise. The coefficient $\phi _{r}$ determines the magnitude of the effect of inter-district spread of contagion, and changes amongst districts according to the population. The third term is the endemic ($\upsilon$) component that determines the district-specific contribution to the number of cases, once the epidemic effects are accounted for. The term $e_{r}$ is the population proportion of district *r*, whereas the term $\upsilon _{r,t}$ consists of a national time trend component, a district-specific effect depending on the share of population, and on a random effect which captures the heterogeneity due to unobserved factors.

Paul and Held (2011) suggested that the endemic and epidemic components can be modelled through the log-linear specifications:

2 $$\begin{aligned} \textrm{log}(\lambda _{rt})=\alpha _{r}^{(\lambda )}+\beta ^{(\lambda )^{\top }}Z_{r,t}^{(\lambda )}, \end{aligned}$$

3 $$\begin{aligned} \textrm{log}(\phi _{rt})=\alpha _{r}^{(\phi )}+\beta ^{(\phi )^{\top }}Z_{r,t}^{(\phi )}, \end{aligned}$$

4 $$\begin{aligned} \textrm{log}(\upsilon _{rt})=\alpha _{r}^{(\upsilon )}+\beta ^{(\upsilon )^{\top }}Z_{r,t}^{(\upsilon )} + \textrm{log}(e_{r,t}), \end{aligned}$$

where the $\alpha _{r}^{(.)}$ is an area-specific intercept *r* and $e_{r,t}$ is the population fraction in area *r* at time *t*. The three main components in Eqs. (2)-(4) describe region-specific effects, incorporated via the use of region-specific random intercepts [18]. In particular, we assume the following normal distributions $\alpha _{r}^{(\lambda )}\sim ^{iid}N(\alpha ^{(\lambda )},\sigma _{(\lambda )}^{2})$, $\alpha _{r}^{\phi }\sim ^{iid}N(\alpha ^{(\phi )},\sigma _{\phi }^{2})$, $\alpha _{r}^{(\upsilon )}$ and $\sim ^{iid}N(\alpha ^{(\upsilon )}, \sigma _{\upsilon }^{2})$. Given the regionally decentralized health system in Rwanda, non-negligible differences in case reporting of COVID-19 infections accross districts are very likely, making inclusion of district-specific intercepts very important.

Both epidemic and endemic components also contain a term $Z_{r,t}^{(.)}$, that represents observed covariates to account for the time trend and to account for the impact of the lockdown that can affect endemic occurrences of infections. More precisely, we fitted models that included lockdown as a dummy variable. The linear Eqs. (5)-(7) show how the parameters were determined.

We assume that the parameter $\lambda _{r,t}$ for the epidemic-within-district component is determined by the following linear equation :

5 $$\begin{aligned} \textrm{log}(\lambda _{r,t})=\alpha _{r}^{(\lambda )}+\beta _{Lockdown}^{(\lambda )}I(Lockdown_{r,t}), \end{aligned}$$

where $\alpha _{r}^{(\lambda )}$ is the random effect intercept, allowed to vary across districts, and $\beta ^{\lambda }_{lockdown}$ is the regression parameter associated with the Lockdown covariate. The lockdown covariate is an indicator equal 1 when lockdown was in place at time t in district r, and 0 otherwise.

For the epidemic-between-districts component, the parameter $\phi _{rc,t}$ is determined by the linear equation:

6 $$\begin{aligned} \textrm{log}(\phi _{r,t})=\alpha _{r}^{(\phi )}+\beta _{pop }^{(\phi )}(pop_{r,t})+\beta _{Lockdown}^{(\phi )}I(Lockdown_{r,t}), \end{aligned}$$

where $\alpha _{r}^{(\phi )}$ is the random effect intercept and $\beta ^{(\phi )}_{lockdown}$ are the regression parameters associated with the population proportion (pop) and lockdown covariates. This model explains the spread of diseases accross different districts. To account for the between-epidemic component three assumptions were tested. We first assumed that the disease transmission can only occur directly from adjacent districts $w_{ji}=\coprod (j\sim i)$, and all districts can equally only have imported cases from neighboring districts. Secondly, we assumed that people travel mainly in urban areas. We adjusted the model to reflect commuter-driven spread in the model, by scaling the district’s susceptibility with respect to its population fraction by multiplying $\phi$ with $e^{\beta _{pop}}$. The last assumption was to consider spatial distance between districts. To account for long-range transmission of cases, we estimated the weights $w_{ji}$ as a function of the adjacency order $O_{ji}$ between the districts. We used a power-law model, that assumes the form value of $w_{ji}=O^{-d}_{ji}$, for $j\ne i$ and $w_{jj}=0$, where $d>0$ is a decay parameter. The greater the d, the faster the decaying of the power low function, and therefore, the less important is the difference between different levels of $O_{ji}$

For the endemic component, the parameter $\upsilon _{r,t}$ is determined by the linear equation:

7 $$\begin{aligned} \textrm{log}(\upsilon _{r,t})=\alpha _{r}^{(\upsilon )}+\beta t+\gamma sin(\omega t)+\delta cos(\omega t) +\beta _{Lockdown}^{(\upsilon )}I(Lockdown_{r,t})+\textrm{log}(e_{r,t}) \end{aligned}$$

Note that the seasonality pattern is included via the sinusoidal wave with frequency $\omega =2\pi /52$.

This model (with normally distributed random effects) can be estimated through penalized likelihood approaches, implemented in the R package surveillance [8]. We implemented the data analysis using the multivariate “hhh4” models in the surveillance package in R software version 4.1.0 [8].

We fitted a model on both all cases (imported and locally transmitted) and only the locally transmitted cases to identify the contribution of imported infections in the model. We compared the basic model (M1), which does not include any covariates. M2 which is the basic model with an added lockdown indicator as a covariate. M3 which builds on M2 by adding population size to account for commuter-driven spread, and M4, which is M3 model adjusted with power-law to account for the long-range transmission of infections.

We introduced random effects to allow district-specific intercepts to improve the model fit. Hence, we updated model (M4) by assuming independent random effects to allow district-specific intercepts to improve the fit (model M5). However, (model M5) does not specify the correlation between the three random effects. We therefore, used a conditional autoregressive formulation (model M6) to account for this correlation.

### Model diagnostics and selection

For the model diagnostics and selection, we used Akaike information criteria (AIC) approaches [19], and predictive model assessment using scoring rules for count data that measure the discrepancy between the predictive distribution from a fitted model and the later observed value. Lower scores correspond to better prediction [8]. The best model was chosen based on predictive model assessment between (model M5) and (model M6) since random effects invalidate comparison of AIC [8]. Table 1 summarize different predictive scores recommended for count data [20].

**Table 1:** Predictive model assessment:

Models	logs	rps	ses
All Cases			
Model M5	18.76	36.41	15405.15
Model M6	18.75	36.41	15391.88
Local cases			
Model M5	18.65	36.24	15470.41
Model M6	17.38	34.51	15343.36

Notes:logarithmic score (“logs”), ranked probability score (“rps”), and squared error score (“ses”). M5 is full model but does not take into account correlation; M6 is full model that account for this correlation

## Results

### Exploratory data analysis

**Fig. 1:**
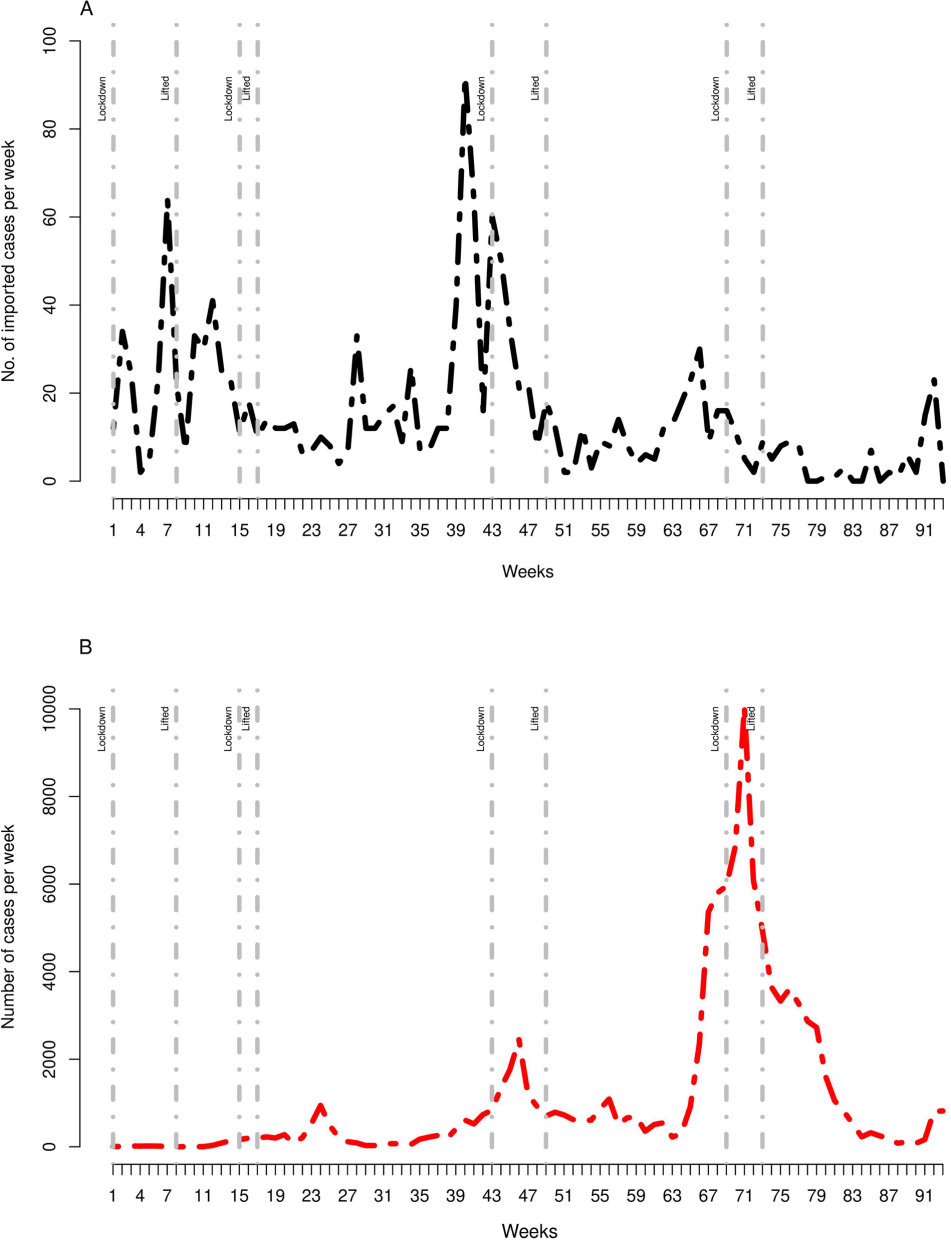
(A) Number of COVID-19 reported cases per week and national lockdown restrictions (grey vertical lines). Top panel: all infected cases in 2020-2021. (B) Lower panel: number of imported COVID-19 cases per week. Notes: The first epidemic week started on $15^{th}$ March 2020, and the last week of the epidemic ends on $19^{th}$ December 2021 in this study

Rwanda recorded the first case of COVID-19 on $14^{th}$ March 2020, which was an imported case from India that triggered the contact tracing efforts. Several other imported cases were identified among travelers, mainly a cluster of business communities who traveled to Dubai and their close contacts in Rwanda [21]. Figure 1 (top panel) shows the trend of the number of COVID-19 reported cases per week for a period of 93 weeks. The first epidemic week started on $15^{th}$ March 2020, and the last week of the epidemic ends on $19^{th}$ December 2021 in this study. The total number of COVID-19 cases in this period was 102,231, including 1,404 imported cases. The graph shows the trends of observed and the imported cases ones. The first wave peak was observed on week 24, the second wave in week 47, and the third wave on week 67. Figure 1 (bottom panel) shows the trend of imported cases over time. There were 15 imported cases per week and 1084 locally transmitted cases per week on average.

Rwanda has had four national lockdowns and several area-specific lockdowns as of December 2021. The first countrywide lockdown started during the period that imported cases were rising and lasted for six weeks (indicated by the grey vertical lines in both the top and bottom panel). The first and second countrywide lockdowns intended to control the rise of cases mainly driven by new imported cases. The third and fourth countrywide lockdowns intended to flatten the curves of the second and third waves. The locally transmitted infections dominated both the third and fourth waves.

**Fig. 2:**
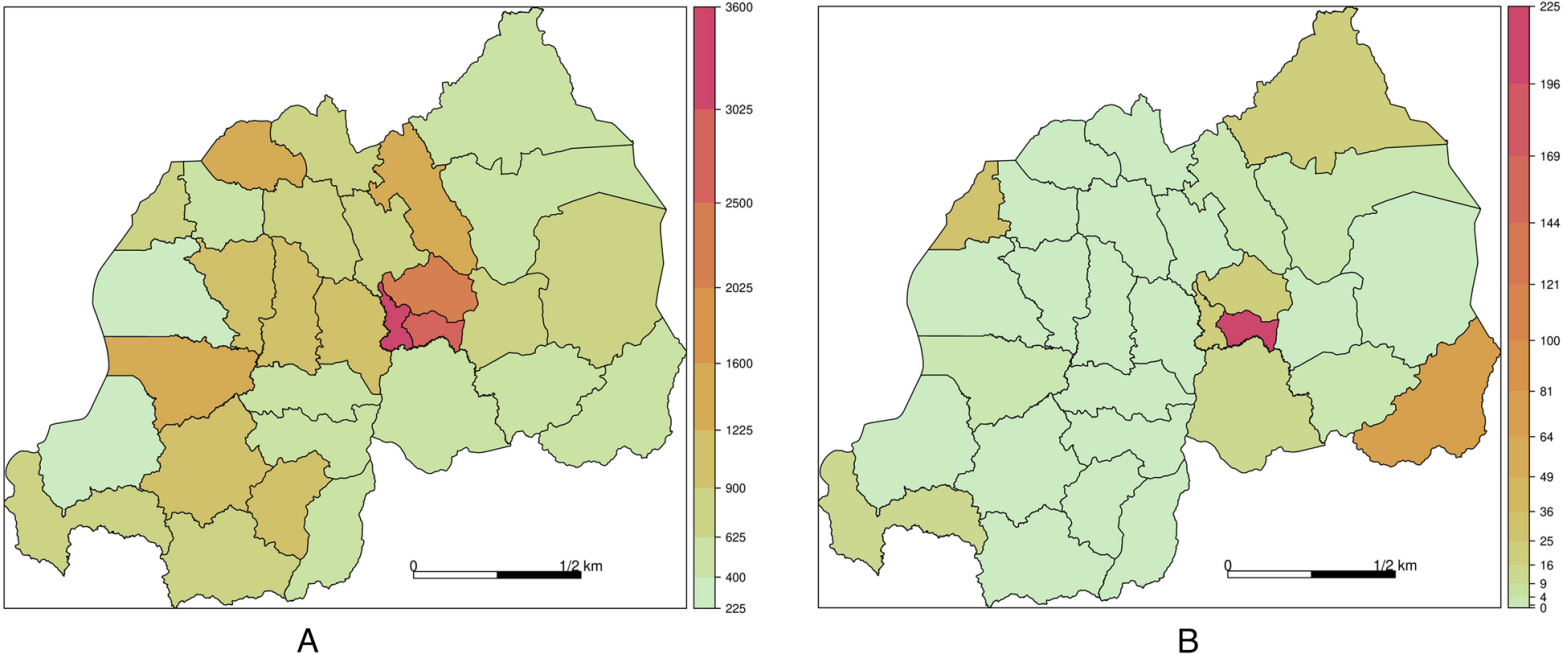
COVID-19 incidence rate per 100,000 inhabitants in Rwanda’s districts. (A) Left: incidence of all cases (local and imported) after 93 weeks. (B) Right: Incidence of imported infected cases after 93 weeks

Figure 2 shows the incidence rate per 100,000 inhabitants per district for all cases (left panel) and for imported cases only (right panel). We observe a high incidence rate of COVID-19 in the central part of Rwanda, mainly in the Gasabo, Kicukiro, and Nyurugenge districts of Kigali city. Furthermore, the left panel of Fig. 2 shows high incidence rates in the South-West (Rusizi) and the South-East (Kirehe) regions. The right panel of Fig. 2 shows high incidence at all points of entry, in the central region of Rwanda (International airport of Kigali), South-West and North-West at the border of Rwanda and Republic Democratic of Congo, and South-East (Kirehe) at the point of entry of Rwanda and the United Republic of Tanzania.

### Main findings

Table 2 shows the distribution of cases by district. The imported cases contributed 1.37% (1,404) of all infected persons, while over 98% of all new infections in Rwanda were locally transmitted. The findings show Kigali City (Kigali city is composed of three districts:Gasabo,Kicukiro and Nyarugenge) and points of entry as the epicenters of COVID-19 in Rwanda. About 43,464 (42.5%) of all infections and 1,355 (96.5%) of imported were found in Kigali and at Rwanda’s borders(Districts at borders:Bugesera,Kirehe,Nyagatare,Rubavu,Rusizi). The city of Kigali contributed 29.48% (30,134) of all infected persons and 57% (803) of all imported infections in Rwanda. About 17% (235) of imported cases were from Tanzania, 12% (172) from Democratic Republic of Congo (DRC), and 7% (101) from Uganda. The results show that eight districts of Rwanda did not record any imported cases and yet contributed about 15% (16,062) of all infected persons in Rwanda. The 1404 imported cases were classified by origin: Asia 200 (14%), Central Africa 138 (10%), East Africa 772 (55%), Europe 87 (6%), Northern Africa 25 (%), Northern America 52 (4%), South Africa 62 (4%), and West Africa 68 (5%).

**Table 2:** Overall COVID-19 infected cases in Rwanda by Districts in 93 weeks

District	n(C%) Imported	n(R%) Imported	n(C%) Local	n(R%) Local
Bugesera	44(3.13)	44 (2.83)	1512 (1.50)	1556 (97.17)
Burera	3(0.21)	3 (0.13)	2317 (2.30)	2320 (99.87)
Gakenke	0(0)	0 (0)	2359 (2.34)	2359 (100)
Gasabo	105(7.48)	105 (0.93)	11206 (11.11)	11311(99.07)
Gatsibo	8(0.57)	8 (0.45)	1755 (1.74)	1763 (99.55)
Gicumbi	7(0.50)	7 (0.13)	5226 (5.18)	5233 (99.87)
Gisagara	0(0)	0 (0)	1738 (1.72)	1738 (100)
Huye	1(0.07)	1 (0.03)	3963 (3.93)	3964 (99.97)
Kamonyi	2(0.14)	2 (0.06)	3176 (3.15)	3178 (99.94)
Karongi	6(0.43)	6 (0.13)	4693 (4.65)	4699 (99.87)
Kayonza	2(0.14)	2 (0.07)	2852 (2.83)	2854 (99.93)
Kicukiro	644(45.87)	644 (7.11)	8416 (8.35)	9060 (92.89)
Kirehe	235(16.74)	235 (11.56)	1798 (1.78)	2033 (88.44)
Muhanga	1(0.07)	1 (0.03)	3412 (3.38)	3413 (99.97)
Musanze	3(0.21)	3 (0.07)	4536 (4.50)	4539 (99.93)
Ngoma	11(0.78)	11 (0.57)	1927 (1.91)	1938 (99.43)
Ngororero	0(0)	0 (0)	3347 (3.32)	3347 (100)
Nyabihu	2(0.14)	2 (0.15)	1310 (1.30)	1312 (99.85)
Nyagatare	101(7.19)	101 (3.56)	2738 (2.72)	2839 (96.44)
Nyamagabe	1(0.07)	1 (0.03)	3178 (3.15)	3179 (99.97)
Nyamasheke	0(0)	0 (0)	1525 (1.51)	1525 (100)
Nyanza	0(0)	0 (0)	2009 (1.99)	2009 (100)
Nyarugenge	54(3.85)	54 (0.55)	9709 (9.63)	9763 (99.45)
Nyaruguru	1(0.07)	1 (0.05)	2169 (99.95)	2170 (99.95)
Rubavu	118(8.40)	118 (3.45)	3303 (3.28)	3421(96.55)
Ruhango	0(0)	0 (0)	1512 (1.50)	1512 (100)
Rulindo	1(0.07)	1 (0.05)	2142 (2.12)	2143 (99.95)
Rusizi	54(3.85)	54 (1.55)	3427 (3.40)	3481 (98.45)
Rutsiro	0(0)	0 (0)	1255 (1.24)	1255 (100)
Rwamagana	0(0)	0 (0)	2317 (2.30)	2317 (100)
**Overall**	**1404 (100)**	**1404 (1.37)**	**100827 (100)**	**100827 (98.63)**

**Table 3:** Model comparison using AIC

	Model All cases		Model Local cases
		AIC	AIC
M1	No covariates	59153.22	58440.21
M2	Lockdown covariate	58837.24	58122.04
M3	Population size and Lockdown	58695.52	57986.63
M4	Power law model	55626.96	55061.39

Notes: M1 is Basic model no covariate; M2 is M1 added Lockdown; M3 is M2 added population size; M4 is M3 update with power law

The model M4 proved to be the best based on AIC as shown in Table 3.

The logarithmic score (logs) and the ranked probability score assess the whole predictive distribution for calibration and sharpness. Then the squared error score is the mean square error of averaged forecasts set. The lower scores correspond to better predictions [20]. The scores for Model M6 are lower as compared to M5 which implies a better model. Therefore the results presented and discussed are based on model M6.

Table 4 shows the parameter estimates for this model. The overall findings of the model show that in Rwanda, within epidemic (autoregressive) contributes 64.2% and 64% with and without imported infections respectively.The infections due to transmission between districts contributes 22.4% and 23% of total spread, correspondingly with and without imported infections. The endemic component contributes respectively 13.4% and 12.5%.

The results in Table 4 show that lockdown contributed to slowing the spread of new infections in both models with and without imported infections, especially with endemic and within spread.

**Fig. 3:**
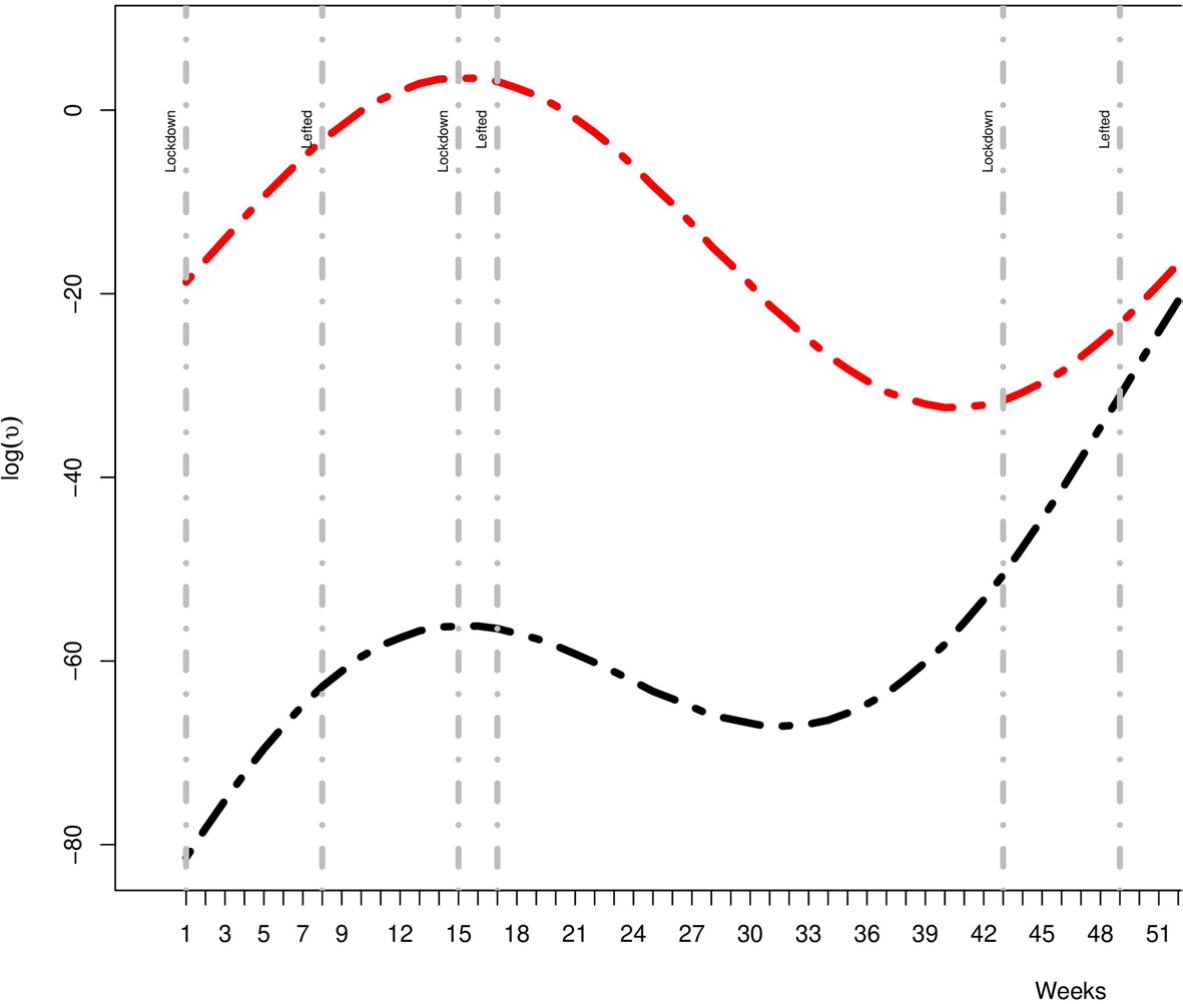
Estimated multiplicative effect of seasonality on the endemic mean and lockdown restrictions(grey vertical dotted lines)

Figure 3 represents seasonality-adjusted factor by which the basic endemic incidence increases per week and lockdown period. The seasonality patterns for both model with and without imported infections are included through sinusoidal wave. The seasonality adjusted factor of the model without imported infection is three times less as compared to model that included all infections. Indeed, the endemic incidence increases 3.5 times $\exp (1.23)$ per week due to imported infections as compared to an increase of 1.1 times $\exp (0.08)$ with only locally transmitted infections. The findings also showed that the amplitude of seasonality is $\sqrt{\gamma +\delta }=4.8$ vs 3.7 without imported infections. This indicate that imported cases do have important influence on the further spread of COVID-19.

**Table 4:** The parameter estimates for final model M6

	**All**		**Local**	
**Parameter**	**Estimates(SD)**	**95% CI**	**Estimates(SD)**	**95% CI**
**Autoregressive**				
$\alpha _{r}^{\lambda }$	-0.50 (0.010)	(-0.52, -0.48)	-0.50 (0.011)	(-0.52, -0.48)
$\beta _{Lockdown}$	-0.36 (0.032)	(-0.42, -0.29)	-0.40 (0.032)	(-0.46, -0.34)
**Spatio-temporal**				
$\alpha _{r}^{\phi }$	-25.42 (7.154)	(-39.44, -11.40)	-21.93 (7.908)	(-37.43, -6.43)
$\beta _{pop}$	1.87 (0.561)	(0.77, 2.96)	1.59 (0.620)	(0.38, 2.88)
$\beta _{Lockdown}$	0.31 (0.044)	(0.22, 0.39)	0.39 (0.041)	(0.31, 0.47)
**Endemic**				
$\alpha _{r}^{\upsilon }$	-107.35 (3.108)	(-113.44, -101.25)	-33.58 (0.500)	(-0.52, -0.48)
$\beta _{t}$	1.23 (0.039)	(1.15, 1.31)	0.08 (0.002)	(0.08, 0.081)
$\gamma$	17.70 (0.577)	(16.57, 18.83)	18.53 (0.465)	(17.61, 19.44)
$\delta$	5.70 (0.237)	(5.23, 6.16)	-4.33(0.106)	(-4.54, -4.12)
$\beta _{Lockdown}$	-0.50(0.076)	(-0.65, -0.35)	-0.62(0.074)	(-0.77, -0.48)
*D*	1.17		1.34	
**Random effect**	**Variance**		**Variance**	
$\sigma _{\lambda }^{2}$	0.31		0.36	
$\sigma _{\phi }^{2}$	0.73		0.91	
$\sigma _{\upsilon }^{2}$	5.01		4.57	
**Contribution of components**				
Autoregressive	64.2%		64.0%	
Spatio-temporal	22.4%		23.5%	
Endemic	13.4%		12.5%	

**Fig. 4:**
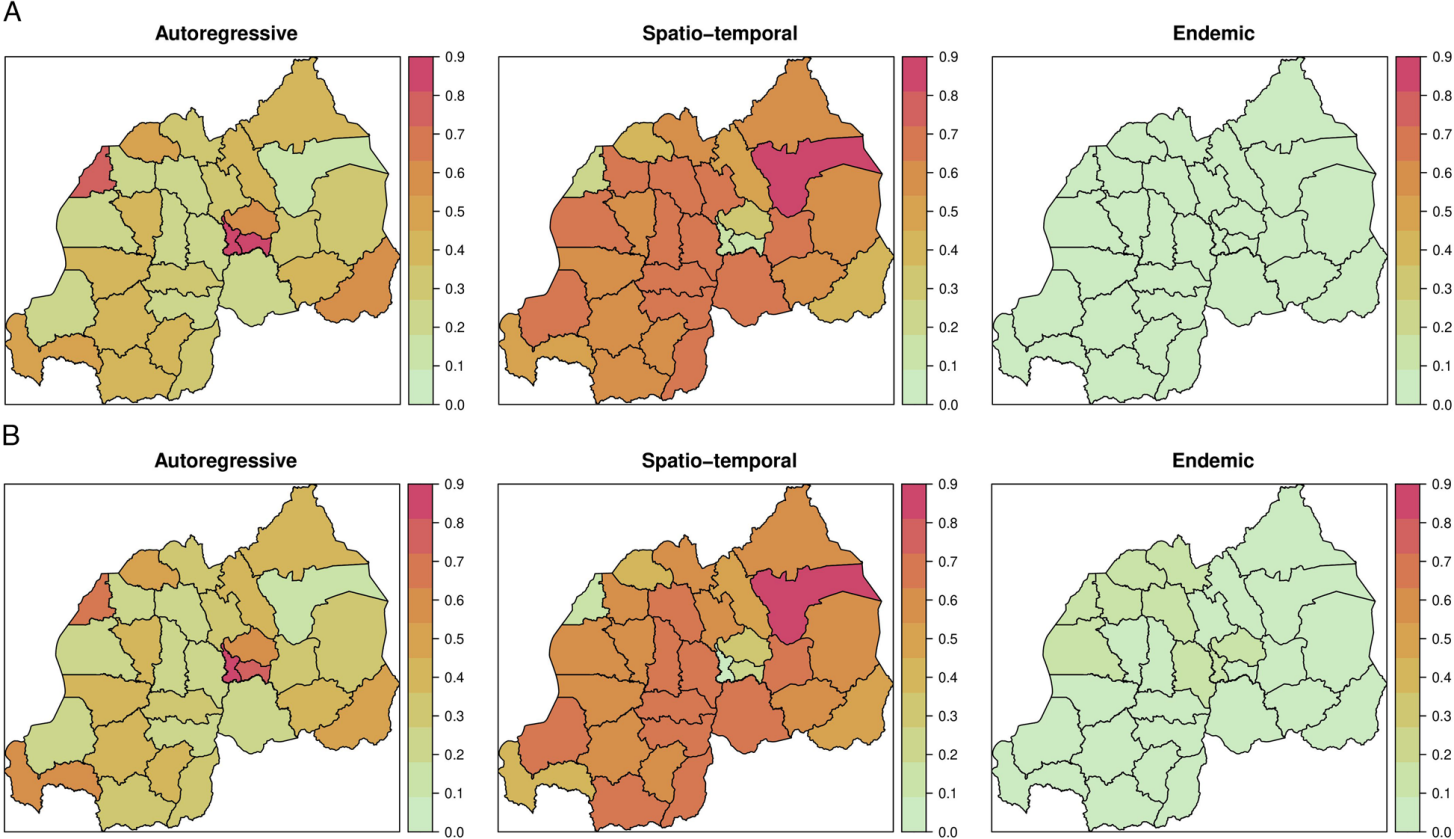
Maps of the fitted component contributions: within epidemic component (Autoregressive), between epidemic component (Spatio-temporal) and endemic component (right). (A) Top panel corresponds to the model for all cases, (B) bottom panel to the model for the local cases

We further assessed the degree of spatial heterogeneity in Fig. 4 for the all cases model (top panel) and for the local cases model (bottom panel). The maps shows the decomposition of the estimated expected number of infections into its three components: the within-epidemic, between-epidemic, and endemic contributions by districts. For each district, the three fitted components are presented as proportions in 4.

Overall, the epidemic within districts (autoregressive) component has the largest contribution in most districts both with and without imported cases, mainly in the three urban districts in central of Rwanda (Gasabo, Nyarugenge and Kicukiro), the district of Rusizi in the South-West, and the district of Kirehe in South East (Map left) and North West (Rubavu). The findings on the autoregressive model with and without imported infections do not show a strong difference. Spatio-temporal model component shows inter-districts transmissions, and the district of Gatsibo in North-East of Rwanda showed a high incidence as compared to other districts. The transmission between neighboring districts is observed in both model with and without imported infections.

Overall findings showed that imported cases have negligible effects due to high domestic transmissibility in Rwanda. The locally transmitted infections are more dominant compared to imported infections. Therefore, non-pharmaceutical interventions focusing on controlling and minimizing the local spread of COVID-19 should be the first priority. Particularly in high incidence parts of the country. A high COVID-19 incidence rate was observed in the central areas of Rwanda, mainly in the three districts of the city of Kigali (Gasabo, Kicukiro, and Nyarugenge), the capital of Rwanda and in districts of Rwanda bordering the United Republic of Tanzania, in Southeast of Rwanda ( Kirehe district), as well as the Democratic Republic of Congo(RDC) in Southwest (Rusizi District) and North West (Rubavu district) as shown in the Fig. 2.”

## Discussion

In this article, we modeled the trend of COVID-19 epidemics accross time and space to understand the dynamics of COVID-19 and to identify the impact of imported cases in Rwanda. Non-pharmaceutical interventions in Rwanda contributed in delaying outbreaks and reduction of new infections. The key Rwanda’s interventions were restricting travels through lockdowns, testing pre and post travels in/out of Rwanda (three polymerase chain reaction (PCR) negatives to all arrivals within 72 hours pre-departure, a second PCR test at arrival, and a third test at the end of 3 or 7 days of quarantine), complete closure of land borders except transportation of goods and other essential travel. In addition to the mandatory policy of wearing the mask, hand washing and other hygienic measures such as sanitizing airport areas, and public places. The transport of people and goods were restricted, except inter-countries truckers of goods. The travel restrictions contributed significantly to keeping imported cases less than 1.5% of all infections in Rwanda. A similarly low number of imported cases were observed in countries with effective screening strategies at the point of entry, such as Beijing in China [22]. About 96.5% (1,355) of imported cases were in urban areas (Kigali City) and the districts at the borders of Rwanda at arrival or through contact tracing. The distribution of confirmed cases is unequal across the Districts. Mainly urban areas are more affected as compared to rural areas [23]. The result showed that 42% of all infections in Rwanda were in Kigali City and areas at the borders of Rwanda and neighboring countries. The urban and the border areas are among the key locations that require specific strategies to control the spread of COVID-19. The paper of Li et al. [22] suggested effective screening strategies at the airport. It is crucial to have effective screening strategies in the framework of preparedness and response to any unexpected public event [24–26]. Unlikely, the paper of Samaan et al. noted the ineffectiveness of screening measures at the point of entry in Australia [27]. Our findings identified the airports areas and the point of entry among hot-spot areas with a high incidence rate compared to the rest of other areas, which justifies the importance of having active Epidemic surveillance at the point of entry.

Appropriate interventions that minimize the spread of infectious diseases such as COVID-19 require a better understanding of epidemic dynamics. The fitted model for the Rwanda case showed that the transmission of disease within the district contributed 64.2% and 64% with and without imported cases. Inter-transmission between districts contributed 22.4% and 23.5% without imported cases. The evolution of the disease over time contributed 13.4% and 12.5% without imported cases. The findings explain the implication of containment strategies applied to control the spread of COVID-19 in Rwanda. The spread of disease between districts was minimized by applying the lockdown strategy, which explains the low contribution (less than 24%) of inter-district transmission. A similar model was used in Italy to understand epidemic dynamics [10]. The non-pharmaceutical interventions, mainly restrictions on movement, border measures, quarantine of travelers arriving from affected countries, city lockdowns, restriction of mass gatherings, isolation and quarantine of confirmed cases and close contacts, social distancing measures, compulsory mask-wearing, contact tracing, and testing, school closures and personal protective equipment use among health workers were effective not only in Rwanda but also in other countries [28][29].

For Rwanda’s situation, although the weekly increase in COVID-19 incidence is much higher with imported infections compared to locally transmitted ones, the impact of imported cases is minimal due to high domestic transmissibility. Therefore, non-pharmaceutical interventions should focus on controlling and minimizing local transmission, especially in high-incidence areas. The study identified several districts with a high incidence rate mainly in urban areas such as districts of the city of Kigali, and districts at point of entry (Kirehe, Rusizi, and Rubavu). Despite the fact that COVID-19 has been spread globally due to international travel and countries have imposed restrictions on travel to curb the spread internally, but the number of new infections did not decrease [30]

The country initiated an in-depth epidemiological investigation, quarantining, testing of contacts, isolation of confirmed cases, mass testing, and vaccine roll-out in order to contain the disease [6]. The mitigation measures were informed by evidence-based decisions [21]. However, there was no available scientific evidence on the effects of imported infections in Rwanda which would help in availing appropriate travel policies and restrictions. Some countries including Rwanda closed their land cross-borders completely, with strict COVID-19 travel restrictions/ban for almost 20 months to prevent imported infections as a mitigation strategy. However, any mitigation strategy should be informed by scientific evidence. Our study provides insight into the effects of imported infections and it reveals the scientific approaches that can be embedded in the existing Rwanda health information system to generate dynamic evidence in real-time. Our findings showed that imported infections have negligible effects on the spread of COVID-19 in Rwanda which implies that re-opening borders and focusing on domestic strategies to control the spread of diseases would be a better strategy to enable the movement of people. The study of Han et al. on quantifying COVID-19 importation risk in a dynamic network of domestic cities and international countries showed that domestic transmissibility interventions are more important than domestic travel flow control [31]. Some papers showed that both domestic and international travel restrictions helped to decrease the confirmed cases and delayed the time to outbreak in the countries with no domestic transmission [32–35]. However, mitigation strategies such as social distancing, testing, contact tracing, and timely quarantine are more effective than travel restrictions in a country that already has community transmission [6, 36].

Managing a pandemic is a complex dynamic process that requires the country to have the ability to distinguish within and between areas of transmission to allow public health experts and policymakers to identify appropriate strategies to prevent the spread of COVID-19. There is no one size fits all, and each country should find the best strategy based on available data or scenario simulations [32]. Still, it is important to have global coordination to ensure the control of pandemics. Rwanda’s response strategies implemented effectively contributed to limiting inter-district transmission and delayed the spread of COVID-19 in the community. Our findings showed that the spatio-temporal component contributes less than 24% in Rwanda’s COVID-19 transmission, which implies that the infection transmission is not neighbor driven. Rwanda adopted non-pharmaceutical interventions such as national and localized lockdown measures with strict enforcement measures to regulate circulation within and between districts to reduce the transmission rate [5]. The lockdown measures succeeded in containing COVID-19 spread in Rwanda, and those measures might be one of the other reasons that infection transmission in Rwanda is not neighbor-driven or there is a low inter-district transmission. On the other hand, lockdown and movement restrictions negatively affected the socio-economic and health sectors [37]. This led to the partial lifting of lockdowns and reluctance to re-imposing them when necessary due to protecting socioeconomic aspects. Therefore, an alternative trade-off strategy that enables early detection of new infections while allowing free movement of people in COVID-19 free environment is needed to maintain the gain from previous measures.

Though imported cases contributed to the spread of COVID-19 at the beginning of the pandemic, imported cases currently have little effect on the COVID-19 pandemic in Rwanda. This study showed that above 98% of infections in Rwanda are locally transmitted. We observed that 15% of all infections in Rwanda were identified in districts that have never recorded an imported case. Once case numbers within the country are sufficiently large due to local transmission travel restriction becomes less effective [16]. Instead, effective screening and necessary isolation measures at the point of entry are crucial for preventing outbreaks that might be caused by imported cases. Countries including Rwanda have restricted international arrivals to prevent the spread of COVID-19. However, those measures carry a high economic and social cost [16].

The paper of Timothy et al. suggested that countries should consider local COVID-19 incidence, local epidemic growth, and travel volumes before implementing travel restrictions [16, 38]. It is helpful for public health decision-making and better planning of detailed public health interventions to understand the dynamics of the epidemic in their specific context.

## Conclusion

In this article, we modeled the trend of the COVID-19 epidemic accross time and space. our use of spatio-temporal modelling enabled us to reproduce the history of the epidemic concerning past strategies put in place by the government of Rwanda to control the spread of COVID-19, allowing us to identify the dynamic evolution of the pandemic for public health guidance.

Our study provides a framework for understanding the dynamics of the epidemic in Rwanda and for monitoring its phenomena to inform timely and targeted interventions.

The findings provide insights into the effects of lockdown and imported infections in Rwanda’s COVID-19 outbreaks. We distinguished between case incidence arising from the local/imported within- or neighbor-driven transmission of infection.

We found that the effect of imported cases was relatively small compared to a sufficiently large local transmission in Rwanda. However, the basic endemic incidence increases triple times per week due to imported cases overtime. Distinguishing within- and between-district transmission of cases allowed us to identify potential strategies for health policy intervention.

Our findings call for using evidence-based strategies to control the spread of COVID-19 and integrating the dynamic models in routine health information management to enable real-time evidence-based decision making.

Based on the results of the study, it is recommended that policymakers should rely on local evidence to develop policies that are tailored to the unique circumstances of their country, rather than adopting global strategies that may not accurately reflect the country’s situation. This approach will ensure that policies are more effective in addressing the specific challenges faced by the country, and will promote better outcomes for the population. These findings have important implications for the development of evidence-based policies that can effectively address the COVID-19 pandemic and other public health crises in a more targeted and efficient manner. Therefore, policymakers are encouraged to consider these recommendations when developing strategies to manage the ongoing COVID-19 pandemic and other public health emergencies.

## Additional file

**Additional file 1.**

## Data Availability

The datasets used during the current study are available from corresponding author on reasonable request.
