# Supplementary figures and images for: Spatio-temporal dynamic of the COVID-19 epidemic and the impact of imported cases in Rwanda
Source: BMC Public Health. 2023 May 23;23:930. doi: 10.1186/s12889-023-15888-1 (PMC10203687; doi:10.1186/s12889-023-15888-1)

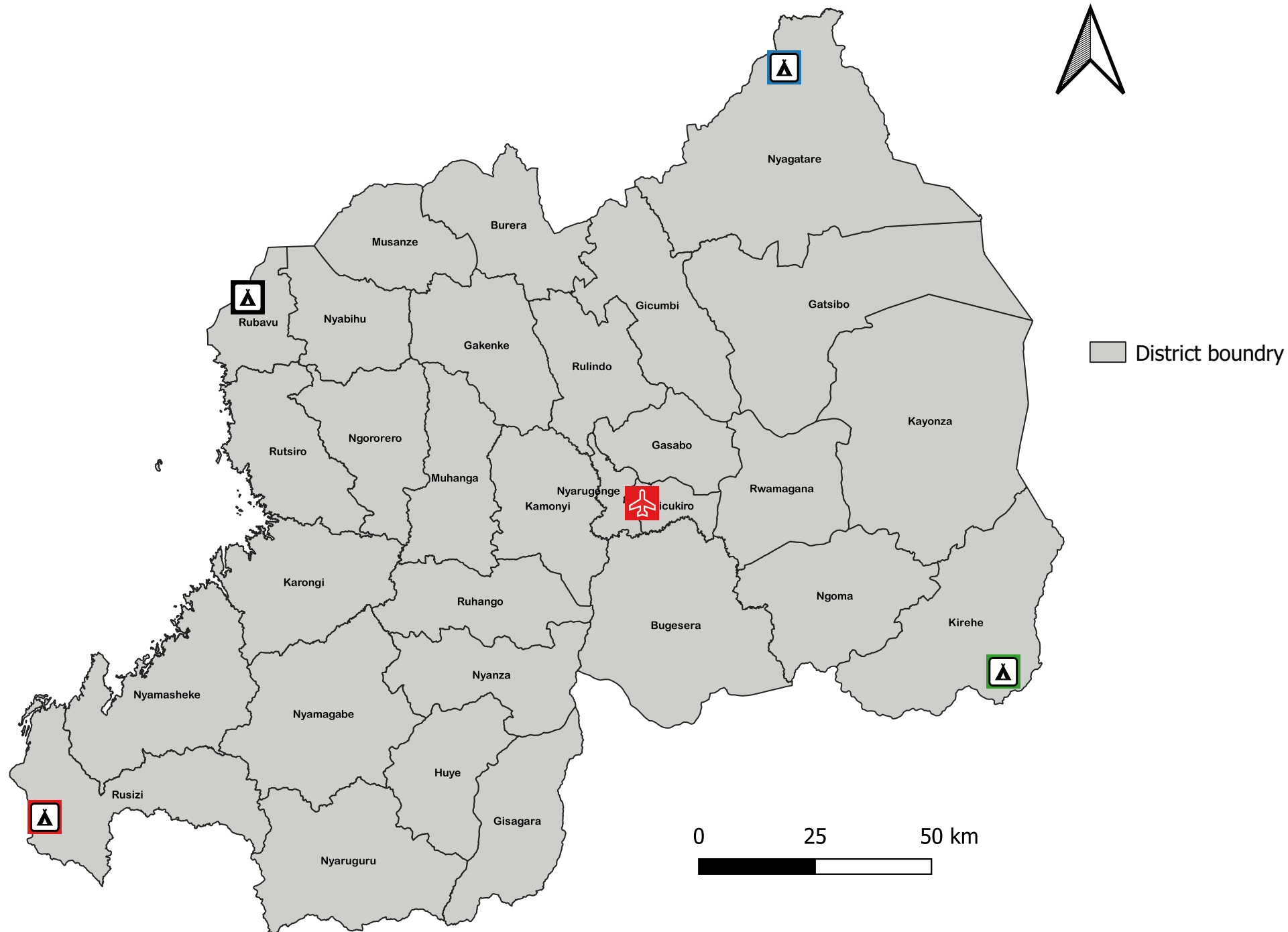

Supplement: Supplementary file 1 — Additional file 1. [file 12889_2023_15888_MOESM1_ESM.pdf]
